# Total Deoxynivalenol Contamination of Wheat Products and Coarse Grains in Shanghai, China: Occurrence and Health Risk Assessment

**DOI:** 10.3390/foods13213373

**Published:** 2024-10-23

**Authors:** Anqi Xu, Shenghao Yu, Yiqi Li, Hong Liu, Zheng Yan, Aibo Wu, Shaojie Peng, Na Liu

**Affiliations:** 1 SIBS-UGENT-SJTU Joint Laboratory of Mycotoxin Research, CAS Key Laboratory of Nutrition, Metabolism and Food Safety, Shanghai Institute of Nutrition and Health, University of Chinese Academy of Sciences, Chinese Academy of Sciences, Shanghai 200031, China; xuanqi2021@sinh.ac.cn (A.X.); zyan@sinh.ac.cn (Z.Y.); abwu@sibs.ac.cn (A.W.); 2 Information Application Research Center of Shanghai Municipal Administration for Market Regulation, Shanghai 200030, China; yshaoyshao@163.com (S.Y.); 18917602951@163.com (Y.L.); 3 Shanghai Municipal Center for Disease Control and Prevention, Shanghai 200336, China; liuhong@scdc.sh.cn

**Keywords:** deoxynivalenol, stable cereals, coarse cereals, contamination, health risk assessment

## Abstract

Deoxynivalenol (DON) is an important mycotoxin produced by *Fusarium* spp., typically found in cereals, which has garnered considerable research attention. However, the risk assessment of DON exposure to muti-cereal is partial and biased, especially lacking the evaluation of different coarse grains. In this study, we synthesized and compared the presence of the total deoxynivalenol (free, acetylated, and masked) of a total of 372 grain samples (17 different types) based on high-performance liquid chromatography–tandem mass spectrometry (HPLC–MS/MS), as well as assessed the chronic and acute risks of total DON exposure in the Chinese population. DON was found at the highest frequency with an occurrence of 85.8% (319/372), followed by D3G at 17.2% (64/372). In total, 88.7% (330/372) of the grains were co-contaminated with mycotoxins. The DON and D3G contamination correlation coefficient was 0.68 in wheat flour. Moreover, different DON contamination levels were found in black beans (133.5 µg/kg), soybeans (128.7 µg/kg), and black rice (122.1 µg/kg). The DON/D3G/15A/tDON contamination level was significant differently among different coarse grains. Notably, the Monte Carlo model showed that 3.2–5.9% of adolescents consuming wheat flour and noodles suffered a chronic tDON risk.

## 1. Introduction

Numerous gramineous crops are susceptible to infection by fungi from the *Fusarium graminearum* species complex (FGSC) [1]. Deoxynivalenol (DON, alias vomitoxin) is a well-known agriculturally important mycotoxin produced by *Fusarium* spp., typically found in cereals, which has garnered considerable research attention due to its high level and frequency of contamination [2,3,4]. Our prior study reported that 85.3% of wheat flour samples (n = 299) were detected to be contaminated with DON and its derivatives [5]. In a survey of 10,192 wheat flours from 30 provinces in China, the average concentration of DON was 250.8 μg/kg [6]. In the field, the DON-producing pathogenic fungus *FGSC* contributed to *Fusarium* head blight (FHB), corn ear rot, and stem rot, which is also related to geographical conditions and climatic factors [7]. Attributed to peculiar climatic conditions, mainly its precipitation and temperature, Shanghai is an area with a high DON contamination concentration in cereals [6]. DON is not easily eliminated in the food processing chain due to its chemical stability. Therefore, cereal products are often contaminated with DON, such as noodles, fermented wheat products, cookies, beer, etc. [8]. Also, 3-acetyldeoxynivalenol (3A-DON), 15-acetyldeoxynivalenol (15A-DON), and deoxynivalenol-3-glucoside (D3G), the main DON derivatives, are frequently detected in cereals. Due to their similar toxicity, a tolerable daily intake (TDI) of 1 µg/kg·bw/day and an acute reference dose (ARfD) of 8 µg/kg·bw/day have been utilized as health guidance values for DON and its derivatives [9,10].

Coarse cereals refer to single-variety commodity cereals and their primary processed products used in domestic consumption or the food industry in China [11]. Wheat flour is one of the main staple grains for Chinese people. Distinguished from staple grains, coarse grains in China include maize, barley, oats, purple rice, whole wheat, buckwheat, rye, various beans, etc., which are similar to whole grains in Western countries, such as Nordic countries, the United States, Australia, and the Mediterranean region [12]. In terms of dietary structure, maize is usually considered as a staple food in some countries, such as Mexico, Central America, and South America. With the high standardization of healthy diets and the benefits of a low glycemic index, elevated dietary fiber content, and superior nutritional profile, continuously more coarse grains have been gradually incorporated into diets, in line with people’s pursuit of a healthy diet [13]. Many other coarse grains are also infested by fungi from the *Fusarium graminearum* species complex (FGSC), the contamination of coarse grains with DON is worth noting. However, the current risk assessment of cereals and their products suffers from the disadvantage of unitary and incomplete coverage, and the risk evaluation and comparison of multi-cereal have rarely been explored, especially lacking the assessment of various coarse grains.

DON exposure in different populations is of concern, especially for adolescents, on account of exceeding the TDI [14,15]. Comprehensive and comparative risk assessments of DON exposure for wheat products and coarse grains are rare in China and worldwide. The research focuses on the following aims: (1) monitoring and comparing the total DON (tDON = DON + D3G + 3A + 15A) contamination in cereals between 2021 and 2023 and investigating the effects of factors such as sample source and type, and (2) surveying the tDON contamination of 5 types of wheat products (staple grain products) and 12 types of coarse grains for the first time, as well as assessing the chronic and acute risk of tDON in five populations (child, teenager, adult, senior, and total population) through the intake of different grains, aiming to provide theoretical guidance and dietary risk data for epidemiological investigations, especially for populations with a high consumption of coarse grains, such as diabetic people and those experiencing weight loss.

## 2. Materials and Methods

### 2.1. Chemicals and Reagents

Chromatographically pure methanol, water, and acetonitrile were purchased from Honeywell, ammonia acetate (Anpel, Shanghai, China) was chromatographic grade, and water was purified by a Genie G5 purifier. The following mycotoxins were purchased from Romer: ^13^C_17_-3-acetyl-deoxynivalenol isotope standard solution (^13^C_17_-3-ADON), ^13^C_15_-deoxynivalenol isotope standard solution (^13^C_15_-DON), DON, 3A-DON, 15A-DON, and D3G. Liquid chromatography with tandem mass spectrometry (8050, Shimadzu, Japan) and an immunoaffinity column (HCM3025B Huaan Magnech Bio-Tech Co., Ltd., Beijing, China) were used throughout the study.

### 2.2. Samples and Sampling

A total of 372 grain samples (17 types) were collected in Shanghai, China, in 2023 and 2024. There are 5 types of wheat flour and products, comprising wheat flour (n = 25), noodles (n = 53), fermented wheat products (n = 28, bread and steamed buns), processed wheat products (n = 76, biscuits, pastries, and puffed-grain food), and beer (n = 25). In total, 12 types of coarse grains were collected, with 10 samples for each type, including barley, oats, mung beans, millet, red beans, glutinous rice, buckwheat, sorghum, maize, black rice, soybeans, and black beans.

The sampling team went to different supermarkets in central and suburban Shanghai to collect samples, then took taxis back to the laboratory. The sample sources were categorized into northern China (NA, n = 65), eastern China (EA, n = 101), central China (CA, n = 87), southern China (SA, n = 69), and foreign regions (n = 50). On the same day, the samples were numbered and recorded with detailed origin information. Most samples were kept in their original packaging, and a few were sampled in bulk. For solid samples, a mixture was taken from the top, middle, and bottom parts of each batch, approximately 150–200 g, and then ground and stored in plastic cans using a grinder. For liquid samples, the samples were taken after thorough mixing and dispensed into three 50 mL centrifuge tubes. All samples were kept at −20 °C prior to analysis.

### 2.3. HPLC–MS/MS Analysis

Firstly, the samples were cleaned by DONS specialized immunoaffinity columns to correct matrix effects [16]. After nitrogen blowing the 5 mL sample extract, 2 mL of water was added for complete dissolution, and then transferred into an immunoaffinity column that was equilibrated to room temperature. A pneumatic pump was integrated with a glass syringe to precisely modulate the flow rate of the sample solution through the immunoaffinity column at a steady pace of one droplet per second, terminating upon the ingress of air into the column. Subsequently, the immunoaffinity column was rinsed sequentially with 5 mL of PBS buffer solution and 5 mL of water. After discarding all the effluent, the column was drained. Then, 2 mL of methanol was added for elution, controlling the dripping rate at one drop per second, and the entire eluate was collected into a test tube. The eluate was dried under nitrogen, followed by the addition of 1.0 mL of the initial mobile phase. The residue was dissolved by vertexing for 30 s, filtered through a 0.22 μm filter membrane, and the filtrate was collected in a sample vial for injection.

The determination method for DONs (DON, 3ADON, and 15ADON) referred to the Chinese standard method (GB5009.111—2016) with minor modifications [16]. The mobile phases consisted of the gradient elution of 100% acetonitrile (B) and 5 mM ammonium acetate water (A) at a flow rate of 0.3 mL/min. The elution schedule was as follows: 10% B for 0–1 min, 10% B for 1–6 min, 90% B for 6–8 min, 90% B for 8–8.1 min, and 90%–10% B for 8.1–10 min. Both positive and negative electrospray ionization were used. A spray voltage of ±3.5 kV, an atomization temperature of 560 °C, a sheath gas pressure of 30 psi, an auxiliary gas pressure of 20 psi, and an ion transfer tube temperature of 300 °C were the settings used for mass spectrometry.

The determination method for D3G referred to the China Grain Industry Standard (LS/T 6133-2018) with minor modifications [17]. For solid samples, 5 g was added to 20.0 mL of extraction solution (acetonitrile/water = 84:16, *v*/*v*), and the mixture was combined with 100 μL of isotope internal standard solution. Then, 1 mL of the supernatant was extracted following 30 min of sonication and 5 min of centrifugation at 4000 rpm. For beer, 5 g of the sample was fixed to a volume of 10 mL with acetonitrile, well mixed, and centrifuged for 10 min at 4000 rpm. After adding 500 µL of the supernatant to 500 µL of water, the mixture was centrifuged for 10 min at 13,000 rpm. Before injection, 20 µL of internal standard solution was mixed with 180 µL of the supernatant. The parameters are shown in Table S1.

### 2.4. HPLC–MS/MS Validation

We performed an estimation of the measurement uncertainty in accordance with the requirements of the China Measurement Standards JJF 1059.1-2012 [18] and CNAS-GL006-2016 [19]. Based on the HPLC–MS/MS isotope internal standard method for the determination of the DON content in the samples [3], the measurement uncertainty was evaluated from the six aspects of sample weighing, analysis instrument, extraction volume, metered volume, sample pretreatment process, and sample repeatability.

The relative combined uncertainty (urel(ω)) and expanded uncertainty (Urel(ω)) for the determination of DON are expressed in Equations (1) and (2):

urel(ω) = ×100%(1)

At a 95% confidence interval, the inclusion factor k = 2 is as follows:

Urel(ω) = urel(ω) × k × 100%(2)

The analytical parameters in the modified HPLC–MS/MS method were analyzed and validated. We used the isotope internal standard method for quantification and correcting matrix effects. The standard curve uses the concentration ratio of the toxin to the internal standard as X and the peak area ratio of target to the internal standard as Y. As for DON, 3A, and 15A, appropriate amounts of mixed standard working solution (DON, 3A, and 15A) and mixed isotopic internal standard working solution (^13^C_15_-DON and ^13^C_17_-3-ADON isotopic internal standards) were accurately pipetted to prepare a mixed standard series with concentrations of 10, 20, 40, 80, 160, 320, and 640 ng/mL, where the concentration of isotopic internal standards was maintained at 100 ng/mL. As for D3G, standard working solution (D3G) and isotopic internal standard working solution (^13^C_15_-DON isotopic internal standards, 50 ng/mL) were mixed to prepare a mixed standard series with concentrations of 10, 20, 40, 80, 160, and 320 ng/mL. The limit of detection (LOD) and limit of quantification (LOQ) are the concentrations at signal-to-noise ratios (S/N) of 3/1 and 10/1 in the sample solution, respectively. The recovery and repeatability of the method were determined by adding different concentrations of mycotoxins to a mycotoxin-free sample with six replicates. The samples were then extracted and analyzed according to Section 2.2 and Section 2.3. The validation parameters are shown in Table S2.

### 2.5. Health Risk Assessment

Risk assessment was conducted by deterministic estimation and probabilistic estimation (Monte Carlo model) [20]. The deterministic assessment model is suitable for screening chemicals, but its limitations include the inability of exposure assessment results to incorporate population variability information under actual dietary exposure scenarios. The probabilistic assessment model describes the likelihood of chemical exposure by constructing a probability distribution of exposure values that can quantitatively describe the variability and uncertainty of a population’s chemical dietary exposure, resulting in a more sophisticated assessment. LOD values were assigned to undetected samples. As shown in Equation (3), probable daily intake (PDI) equals the average contamination level multiplied by the population consumption divided by body weight [21]. The demographic and dietary intake parameters evaluated in the present investigation align with those reported in prior research endeavors [22,23,24].

PDI = (C × CA)/Bw(3)

where:

C = mean concentration of mycotoxins (μg/kg)
CA = consumption data (g·person^−1^·day^−1^)
Bw = body weight (kg)

The consumption data were based on the daily dietary intake of various food groups of Shanghai residents aged 15 years and above surveyed by the Shanghai Municipal Centre for Disease Control and Prevention (SMCDC) [24] and the 2013 Shanghai Food Consumption Survey (SFCS) [22].

### 2.6. Data Analysis

Means ± standard errors are used to express the data. At a significance level of 0.05, non-parametric tests such the Mann–Whitney and Kruskal–Wallis tests were performed to compare the distributional features and variations between samples. Plotting and data management were handled entirely with GraphPad Prism 9.0 (San Diego, CA, USA). For samples where no detection was achieved (below the limit of detection, <LOD), values of 0, half the LOD (1/2 LOD), and the LOD were adopted as substitutions for the lower, median, and upper bounds, respectively, in accordance with the previous literature [23]. The primary focus of this study was on the data pertaining to the upper bound. Considering the low detection rate of masked mycotoxins, values are uniformly assigned in accordance with the limit of detection (LOD) based on the principle of the maximum protection of the population.

## 3. Results and Discussion

### 3.1. Method Validation

The method was validated in terms of linearity, correlation coefficients (R^2^), limit of determination (LOD), limit of quantification (LOQ), and recovery (Table S2). In order to correct matrix effects, we used DONs immunoaffinity columns for purifying the samples and the isotope internal standard (13C15-DON and 13C17-3-ADON isotopic internal standards) method for quantification [16]. The detection value we obtained was for absolute quantification rather than relative quantification, therefore, our detection data were totally accurate and reliable. As for linearity, the correlation coefficients (R2) of DON and its derivatives were determined to range from 0.9934 to 0.9998 [25]. After optimization, an LCMS 8050 system (Shimadzu, Japan) equipped with a waters BEH-C18 Column (2.1 ∗ 150 mm,1.7 μm) was used. The recovery of each target analyte was measured at three spiked concentrations based on blank solid (wheat flour) and liquid sample (beer) matrices. The recoveries ranged from 81.2 to 123.6% with an RSD of less than 20%, which is in accordance with the guidelines of the document [26,27]. The expended uncertainty result was 0.93 µg/kg (k = 2) for a DON addition level of 10 µg/kg (n = 6). The main sources affecting uncertainty were the repeatability tests and analytical instrumentation. Similarly, the extended uncertainties of measurement for 3A, 15A, and D3G were 1.01, 1.04, and 0.95 µg/kg (k = 2) for the mycotoxin level of 10 µg/kg (n = 6), respectively. Taken together, the validation results indicate that the modified method could be applied for the quantitative determination of DON and its derivatives in wheat products and coarse grains.

### 3.2. Differences in Mycotoxin Occurrence in 2021 and 2023

Mycotoxin formation is influenced by many factors, such as climate and storage. Our research group compared different harvest years for wheat flour and products in 2021 and 2023, as shown in Table 1 and Table S3. Compared with our previous work (299 wheat flour samples) in 2021, we increased the sample types (17) and sample numbers (372) in 2023. The sample types were increased from just wheat flour to whole grains and their products. Thus, a comprehensive picture of the contamination status of DON and its derived mycotoxins in different cereals and their products was made clear.

Firstly, we focused on the differences in mycotoxin occurrence in 2021 and 2023. The mean concentrations of DON (154.8 µg/kg) and 15A (5.0 µg/kg) in wheat flour in 2023 were significantly higher than those in our 2021 work (DON 57.6 µg/kg, 15A 1.7 µg/kg) [5]. The mean concentrations of D3G (12.0 µg/kg) and tDON (176.9 µg/kg) in 2023 were higher than those in 2021 for wheat flour (D3G 8.6 µg/kg, tDON 79.8 µg/kg). The FGSC grows most rapidly at temperatures between 25 and 28 °C with a humidity level above 90%. Wheat crops are most susceptible to infection by the FGSC and the production of DON from the flowering stage (anthesis) to the early milk stage during April and June. The weather was different between 2021 and 2023, mainly due to temperature and humidity. In 2021, the average temperatures in May typically ranged from around 15 °C to 24 °C. In 2023, the overall temperatures in May (20–30 °C) were reported to be slightly higher than those in in 2021. The rainfall in 2023 was significantly higher than that in 2021, especially during May and June. This combination of higher temperatures and increased humidity in 2023 likely led to higher DON production. No samples were found to exceed the limit value (1000 µg/kg) [16]. Yu et al. [22] also reported differences in the DON contamination levels in wheat flour and its products from 2017 to 2021. Wheat contaminated with DON varies from year to year depending on rainfall and temperature. The contamination rate and levels of DON in wheat flour vary in different countries, as follows: Egypt in 2022 (56%, 188 μg/kg) [28], Türkiye in 2020 (6%, 116.3 μg/kg) [29], and Korea (96%, 38.1 μg/kg) [30]. Next, we conducted a specific contamination analysis of samples in 2023 to explore the effects of sample origin, type, coexistence, and other factors.

### 3.3. Mycotoxin Occurrence in Total Samples

**Figure 1:**
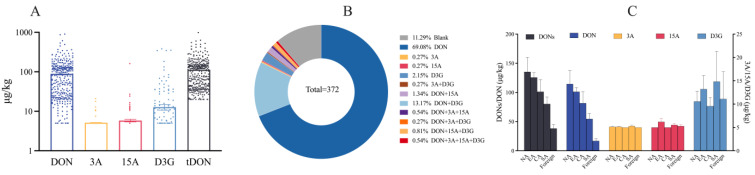
Contamination of detected mycotoxins in total cereals and their products (µg/kg, n = 372) (**A**) Contamination levels in total samples (the top line of the bar graph represents the mean); The dots means the values of samples. (**B**) co-occurrence of mycotoxins in total samples; (**C**) geographical distribution of mycotoxins in total samples (NA: Heilongjiang, Jilin, Liaoning, Inner Mongolia, Beijing, Hebei, and Tianjin; EA: Shandong, Shanghai, Jiangsu, Zhejiang, Anhui, and Fujian; CA: Henan, Hubei, and Hunan; SA: Guangxi, Guangdong, Sichuan, Guizhou, and Yunnan; and Foreign: Japan, Italy, Thailand, Germany, South Korea, and Indonesia).

Processing and sample type had significant effects on the toxin levels in cereals and their products [31,32,33,34]. A total of 372 grain samples were selected, as shown in Figure 1. As shown in Table 1 and Figure 1A, the average contamination levels of DON, 3A, 15A, and D3G were 88.9, 5.2, 5.8, and 12.8 µg/kg and the maximum values were 902.1, 21, 161.5, and 381.5 µg/kg, respectively. The average contamination level of DON in our study was lower than that reported by some scholars, as follows: Gab-Allah (188 µg/kg) [28], Wang (250.8 µg/kg) [6], and Yang (205.6 µg/kg) [35]. DON had the highest positivity rate of 85.8% (319/372), followed by D3G (17.2%), 15A (3.8%), and 3A (2.1%). tDON (DON + D3G + 3A + 15A) had an average contamination level of 112.6 µg/kg, and one maize flake sample had a maximum contamination level of 1076 µg/kg.

The co-occurrence of mycotoxins in cereals and their products is illustrated in Figure 1B. In total, 42 samples (11.3%) were negative. A total of 71.8% of the samples were contaminated with one mycotoxin, while 14.8% and 1.6% of the samples were contaminated with two and three mycotoxins, respectively. Only two samples were contaminated by four mycotoxins (DON + 3A + 15A + D3G). The frequency of DON + D3G was 13.2%, followed by D3G (2.2%), DON + 15A (1.3%), and DON + D3G + 15A (0.8%). The simultaneous presence of multiple mycotoxins was different from that recorded by Zhao et al. [36]. The co-occurrence of mycotoxins in cereals has frequently reported [37].The co-occurrence type of DON + D3G should be considered for regulation.

The sample sources were categorized into northern China (NA), eastern China (EA), central China (CA), southern China (SA), and foreign regions (Figure 1C). The average contamination levels of tDON and DON were the highest in NA. The mean contamination levels of tDON and DON followed the same trend, as follows: NA (135.3, 114.5 µg/kg) > EA (125.8, 101.2 µg/kg) > CA (101.2, 81.6 µg/kg) > SA (80.4, 54.7 µg/kg) > Foreign (38.3, 16.7 µg/kg). The detection rates of DON in different regions were as follows: EA 90.9% > NA 84.1% > CA 82.1% > SA 79.7% > foreign 59.1%. The average contamination levels of D3G, in descending order, were as follows: SA 14.8 µg/kg > EA 13.2 µg/kg > Foreign 11.1 µg/kg > NA 10.6 µg/kg > CA 9.6 µg/kg. There was a minor difference between the contaminant levels of 3A and 15A. The contamination levels of tDON and DON in NA were higher than those in other regions, and these results were similar to previous reports [6,35]. Mycotoxin contamination is related to many factors such as geography, cropping practices, weather factors, climatic factors, soil environment, and climatic and rainfall conditions during wheat cultivation that favor *Fusarium* spp. infection and DON synthesis. The plant status is determined by the interplay of plant–pathogen–microbiota in the rhizosphere [38]. Cropping practices affect soil acidification, the accumulation of autotoxic substances, and root-associated microbiota [39], which may explain the contamination differences between different areas.

#### 3.3.1. Mycotoxin Occurrence in Detailed Wheat Products

**Figure 2:**
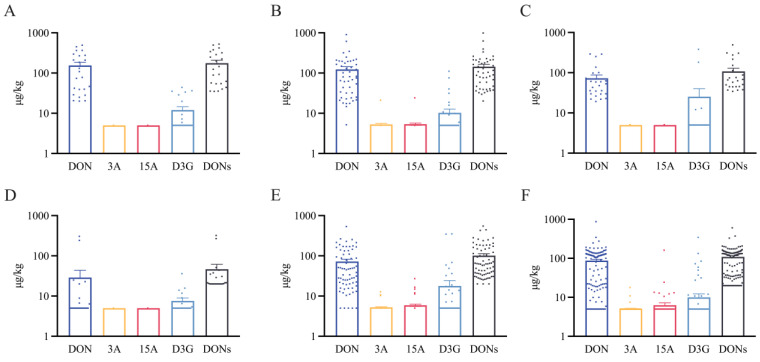
Contamination of detected mycotoxins in different cereals and their products. (**A**) Wheat flour; (**B**) noodles; (**C**) fermented products; (**D**) beer; (**E**) processed products; and (**F**) coarse grains. The dots means the values of samples.

Processing had a significant effect on the toxin levels in wheat end products [31,32]. Figure 2 and Table S3 present the contamination levels of the detected mycotoxins in different types of cereals and their products. The contamination levels of DON, D3G, and tDON in different kinds of samples were significantly different (*p* < 0.05 for Kruskal–Wallis rest). The mean contamination levels of DON were ranked as follows: wheat flour (154.8 µg/kg) > noodles (123.1 µg/kg) > coarse grains (87.3 µg/kg) > fermented products (72.9 µg/kg) > processed products (72.6 µg/kg) > beer (28.9 µg/kg). tDON showed the same trend. The DON and tDON contamination levels were significantly higher in wheat flour and noodles, fermented products, and processed products than in beer. Moreover, it is noteworthy that the positivity rates of DON were as follows: wheat flour = noodles = fermented products (100%) > processed products (92.1%) > coarse grains (81.2%) > beer (36%). In Beirut, the mean contamination data for DON were as follows: fermented wheat product (bread 176 µg/kg) and processed products (biscuits 31 µg/kg, cakes 60 µg/kg) [33]. The average contamination levels of D3G were as follows: fermented products (25.3 µg/kg) > processed products (17.9 µg/kg) > wheat flour (12.0 µg/kg) > noodles (10.3 µg/kg) > coarse grains (9.9 µg/kg) > beer 7.6 µg/kg. Wheat flour contained significantly higher levels of D3G than coarse grains. The D3G positivity rates in descending order were as follows: wheat flour (40%) > beer (32%) > processed products (21.1%) > noodles (20.8%) > fermented products (14.3%) > coarse grains (9.1%). Interestingly, the correlation coefficient between the DON and D3G contamination of wheat flour was 0.68 (Figure S1A). DON would decrease after processing wheat flour into wheat products, and our findings are similar to previous reports [40,41,42,43]. In addition, the highest D3G level was found in fermented wheat products (25.3 µg/kg), which may be attributed to fermentation favoring the transformation of toxins [44].

#### 3.3.2. Mycotoxin Occurrence in Detailed Coarse Grains

**Figure 3:**
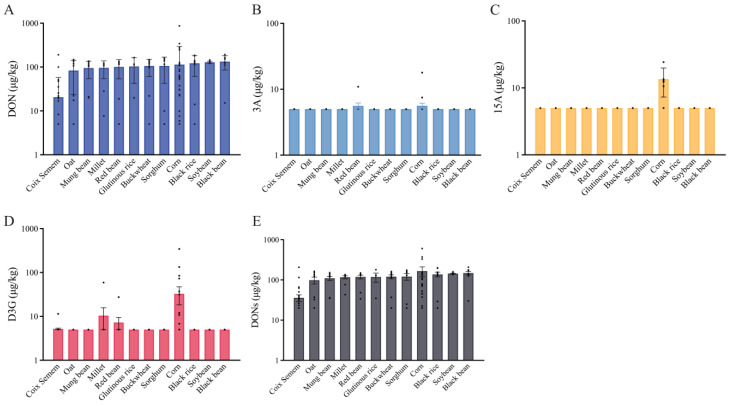
Contamination of detected mycotoxins in 12 types of coarse grains (µg/kg, n = 165). The dots means the values of samples. (**A**) DON contamination in coarse grains; (**B**) 3A contamination in coarse grains; (**C**) 15A contamination in coarse grains; (**D**) D3G contamination in coarse grains; (**E**) DONs contamination in coarse grains.

Numerous coarse grains are also infested by mycotoxins. However, current studies on the determination of coarse cereals are rare and incomplete, mostly focusing on maize, barley, and coix seeds [21,30]. We encompassed and monitored broader species of coarse grains (12 species) in this study, as shown in Figure 3. The contamination levels of DON, 15A, D3G, and tDON in different types of coarse grains were notably different (*p* < 0.05 for Kruskal–Wallis test), while that of 3A was not significantly different. The average contamination levels of DON in different types of coarse grains were ranked in descending order as follows: black bean (10/10, 133.5 µg/kg) > soybean (10/10, 128.7 µg/kg) > black rice (10/11, 122.1 µg/kg) > maize (23/25, 113.9 µg/kg) > buckwheat (11/12, 105.7 µg/kg) > sorghum (7/8, 105.7 µg/kg) > glutinous rice (5/5, 103.3 µg/kg) > red bean (9/10, 101.1 µg/kg) > millet (10/10, 96.1 µg/kg) > mung bean (10/10, 95.0 µg/kg) > oats (9/10, 83.6 µg/kg) > seed of *Coix* spp. (11/31, 20.6 µg/kg). In terms of dietary structure, maize is considered to be a staple food in other countries, such as Mexico. The average contamination levels of 15A and D3G in maize were significantly higher than others. The highest mean contamination level of tDON was found in maize and the lowest in seeds of *Coix* spp. Some studies have reported the contamination of DON in coarse grains as follows: China [21], seed of *Coix* spp. (9/60, 77.2 µg/kg); Finland [34], oat (4/7, 29.9 µg/kg), barley (1/9, 3.9 µg/kg), and rye (5/11, 23.2 µg/kg); Korea [45], glutinous rice (1/11, 1.7 µg/kg), brown rice (16/48, 7.1 µg/kg), and barley (22/39, 16.4 µg/kg); and Nigeria [46], sorghum and millet (not detected). Notably, we found high contamination levels of DON in black beans (10/10, 133.5 µg/kg), soybeans (10/10, 128.7 µg/kg), and black rice (10/11, 122.1 µg/kg), which had not been previously reported.

### 3.4. Risk of Chronic and Acute DON Exposure

**Figure 4:**
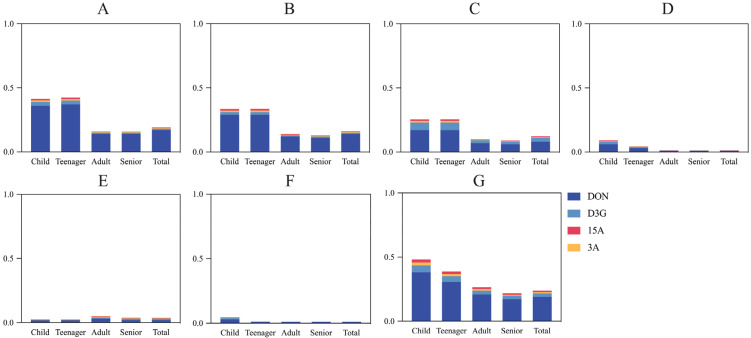
Probable daily intake estimated from chronic tDON exposure in different cereals and their products for populations performed by deterministic estimation (µg/kg·bw/day). (**A**) Wheat flour; (**B**) noodles; (**C**) fermented products; (**D**) beer; (**E**) processed products; (**F**) coarse grains; and (**G**) total samples.

**Figure 5:**
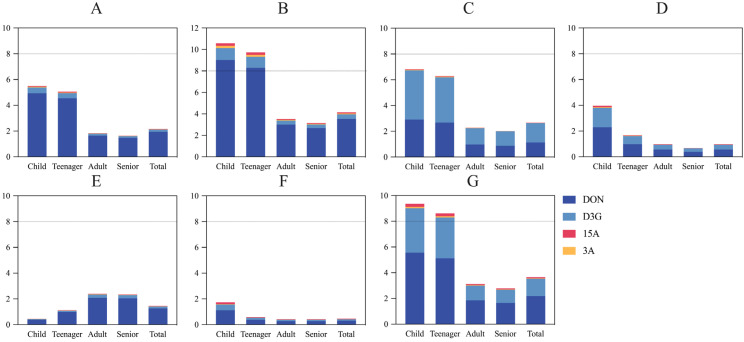
Probable daily intake from acute tDON exposure in different cereals and their products for populations performed by deterministic estimation (µg/kg·bw/day). (**A**) Wheat flour; (**B**) noodles; (**C**) fermented products; (**D**) beer; (**E**) processed products; (**F**) coarse grains; and (**G**) total samples. The dashed line means the ARfD value.

**Figure 6:**
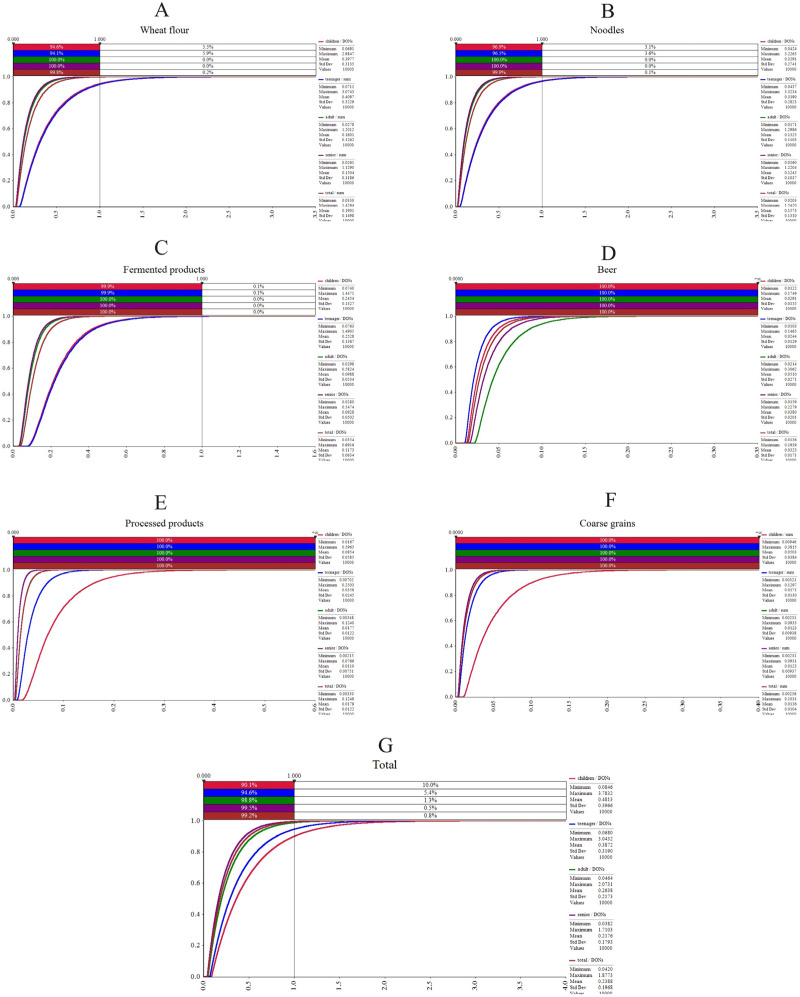
Probable daily intake intake from tDON exposure in different cereals and their products for populations performed by probabilistic estimation (µg/kg·bw/day). (**A**) Wheat flour; (**B**) noodles; (**C**) fermented products; (**D**) beer; (**E**) processed products; (**F**) coarse grains; and (**G**) total samples.

The total samples were subdivided into six categories. We performed chronic and acute exposure risk assessments of tDON by deterministic estimation and probabilistic estimation (Figure 4, Figure 5 and Figure 6).

First, we performed chronic risk assessments. In terms of deterministic estimation, the PDI values were less than the TDI values (1 µg/kg·bw/day) (Figure 4), indicating no chronic tDON risk for five populations consuming cereals and their products.

In terms of probabilistic assessment, there were 10.0% of children, 5.4% of teenagers, and 1.3% of adults who consumed overall cereals and their products suffering from a chronic tDON risk (Figure 6G). In detaile, 5.4% and 3.2% of children, as well as 5.9% and 3.6% of teenagers, were at a chronic risk from consuming wheat flour and noodles, respectively (Figure 6A,B). None of the processed products, beer, and coarse grains posed a chronic tDON risk based two estimations.

Next, we conducted acute risk assessments. Typically, P99 is used to indicate acute toxicity exposure risk [47]. Point assessment showed that children and teenagers’ intake of noodles and the total samples put them at risk for acute tDON exposure, as the PDI values exceeded the ARfD value (8 µg/kg·bw/day) (Figure 5B,G). The modeling assessment showed that the five populations were safe. The point assessment was slightly overestimated compared to the modeling assessment.

After that, we performed the population risk ranking. In most cases, the population risk ranking was as follows: teenager > child > total > adult > senior. However, adults were most at risk for drinking beer compared with other populations (Figure 4E, Figure 5E and Figure 6E). To summarize, children and teenagers suffer from a tDON exposure risk through their intake of wheat flour and noodles. Teenagers and children were more frequently exposed to risk in our study due to their high food intake relative to their body weight. Previous studies are consistent with our study, which have also found that children are exposed to higher amounts of DON per day than adults [6,14,20,48]. The health of the children and teenager populations is particularly worth monitoring.

## 4. Conclusions

In the present study, 17 different types of cereals and their products (372 samples) were investigated, and DON was the abundant mycotoxin. DON was found at the highest frequency with an occurrence of 85.8% (319/372), followed by D3G at 17.2% (64/372). The contamination correlation coefficient value of wheat flour was 0.68 between DON and D3G. The co-occurrence type of DON + D3G (13.2%) should be considered for regulation. Among the coarse grains, higher DON contamination levels were found in black beans (133.5 µg/kg), soybeans (128.7 µg/kg), and black rice (122.1 µg/kg), which deserve concern and further studies. Notably, 3.2–5.9% of adolescents consuming wheat flour and noodles suffered from a chronic tDON risk based on the Monte Carlo model. Research on the determination and evaluation of muti-mycotoxins in diverse coarse grains should be augmented. The examination of coarse grains in this study provides background data on the toxins in different grains and offers a dietary risk profile for people who replace staple foods with coarse grains.

## Figures and Tables

**Table 1:** Comparison of detected mycotoxins in cereals and their products in 2021 and 2023 (µg/kg).

Year	Sample	Number	Toxin	Positive Rate	Over MRL	Mean	P50	P95	Max	Reference
2021	Wheat flour	299	DON	74.9	0.0	57.6	34.4	190.0	371.4	[5]
			3A	4.0	0.0	12.0	0.8	53.1	140.6	
			15A	37.8	0.0	1.7	1.6	1.6	10.8	
			D3G	32.1	0.0	8.6	1.6	43.0	96.3	
			tDON	85.3	-	79.8	56.3	251.1	422.0	
2023	Grains and products	372	DON	85.8	0.0	88.9	52.3	265.4	902.1	This work
			3A	2.1	0.0	5.2	5.0	5.0	21.0	
			15A	3.8	0.0	5.8	5.0	5.0	161.5	
			D3G	17.2	0.0	12.8	5.0	36.6	381.5	
			tDON	92.4	-	112.6	75.2	320.0	1076	

## Data Availability

The original contributions presented in the study are included in the article/Supplementary Materials, further inquiries can be directed to the corresponding authors.

## Supplementary Materials

The following supporting information can be downloaded at: https://www.mdpi.com/article/10.3390/foods13213373/s1, Table S1. LC-MS/MS analytical parameters for the analysis of mycotoxins; Table S2. Overview of the method validation parameters including linearity range, correlation coefficient (R^2^), limits of detection (LOD), limits of quantification (LOQ), and recovery for the analysis of mycotoxins; Table S3. Contamination of detected mycotoxins in different cereals and their products for dietary exposure assessment in 2023 (µg/kg, n = 372); Figure S1. Correlation coefficients of detected mycotoxins in different cereals and their products. (**A**) Wheat flour; (**B**) noodles; (**C**) fermented products; (**D**) beer; (**E**) processed products; and (**F**) coarse grains.
