# Supplementary material for: Total Deoxynivalenol Contamination of Wheat Products and Coarse Grains in Shanghai, China: Occurrence and Health Risk Assessment
Source: Foods. 2024 Oct 23;13(21):3373. doi: 10.3390/foods13213373 (PMC11545617; doi:10.3390/foods13213373)
Supplement: Supplementary file 1 [file foods-13-03373-s001.zip › foods-3220978-supplementary.pdf]

Supplementary data

Table S1. LC-MS/MS analytical parameters for the analysis of mycotoxins.

Table S2. Overview of the method validation parameters including Linearity range, correlation coefficient (R^2), limits of detection (LOD); limits of quantification (LOQ) and recovery for the analysis of mycotoxins

Table S3. Contamination of detected mycotoxins in different cereals and their products (μg/kg, n=372).

Figure S1. Correlation coefficient of detected mycotoxins in different cereals and their products. A: wheat flour; B: noodles; C: fermented products; D: beer; E: processed products; F: coarse grains.

Table S1. LC-MS/MS analytical parameters for the analysis of mycotoxins.

Mycotoxins	Molecular Weight	RT (min)	Molecular Ion	ESI	Parent Ion (*m*/*z*)	Product Ions (*m*/*z*)	CE (eV)
DON	296.32	3.0	$[M - H]^-$	-	295.3	265*/138	12/24
D3G	458.50	4.6	$[M+CH3C00]^-$	-	517.5	427.4*/457.4	22/14
3A-DON	338.35	4.6	$[M-H]^-$	-	337.3	307*/173	10/12
15A-DON	338.35	4.6	$[M-H]^-$	-	337.3	150*/239	12/12
^{13}C-DON	311.32	3.0	$[M - H]^-$	-	310.3	279*/145	12/14
^{13}C-3-ADON	355.35	4.6	$[M - H]^-$	-	354.3	323*/230	14/18

Note: 15-ADON and D3G, ^{13}C-3-ADON was chosen as the isotopic internal standard for the corresponding quantitative calculations.

*: quantitative ion

Table S2. Overview of the method validation parameters including linearity range, correlation coefficient (R^2), limits of detection (LOD); limits of quantification (LOQ) and recovery for the analysis of mycotoxins.

Mycotoxin	Linearity Range (μg/kg)	Regression equation	Correlation Coefficient (R^2)	LOD (μg/kg)	LOQ (μg/kg)	Spiked Sample	Recovery ± RSD (%, n = 6)		
							Spiked Low	Spiked Middle	Spiked High
DON	10-640	Y=0.0166935x+0.0144443	0.9997	5	10	wheat flour	98.2±2.5	106.2±1.7	107.5±1.3
						beer	110.7±3.3	104.2±2.0	98.0±1.7
D3G	10–320	Y=0.0147x+0.0879	0.9996	5	10	wheat flour	99.9±4.6	98.2±4.6	96.7±4.2
						beer	123.6±7.1	81.2±5.2	105.1±3.9
3A-DON	10-640	Y=0.0187806x+0.0414993	0.9996	5	10	wheat flour	95.5±5.8	97.7±3.5	96.9±3.6
						beer	89.9±2.9	85.9±2.6	96.8±1.4
15A-DON	10-640	Y=0.00508783x-0.00165153	0.9993	5	10	wheat flour	94.8±3.2	90.7±1.3	88.5±1.0
						beer	90.7±5.3	96.9±2.1	80.8±1.3

Table S3 Contamination of detected mycotoxins in different cereals and their products for dietary exposure assessment in 2023 (μg/kg, n=372).

		Wheat flour	Noodles	Fermented product	Processed product	Beer	Coarse grains
	Number	25	53	28	76	25	165
DON	Positive rate/%	100	100	100	92.1	36	81.2
	Mean	154.8	123.1	72.9	72.6	28.9	87.3
	Max	494.2	902.1	291.4	536.5	306.0	870.1
3A	Positive rate/%	0	1.9	0	3.9	0	1.8
	Mean	< LOD (5.0)	5.3	< LOD (5.0)	5.2	< LOD (5.0)	5.1
	Max	< LOD (5.0)	21.0	< LOD (5.0)	12.8	< LOD (5.0)	17.9
15A	Positive rate/%	0	1.9	0	7.9	0	3.0
	Mean	< LOD (5.0)	5.4	< LOD (5.0)	5.9	< LOD (5.0)	6.3
	Max	< LOD (5.0)	24.2	< LOD (5.0)	27.1	< LOD (5.0)	161.5
D3G	Positive rate/%	40	20.8	14.3	21.1	32	9.1
	Mean	12.0	10.3	25.3±14.6	17.9	7.6	9.9
	Max	44.0	110.8	381.5	352.6	36.1	344.2

Note: The values for undetected (<LOD) samples were substituted with half of the corresponding LOD value.

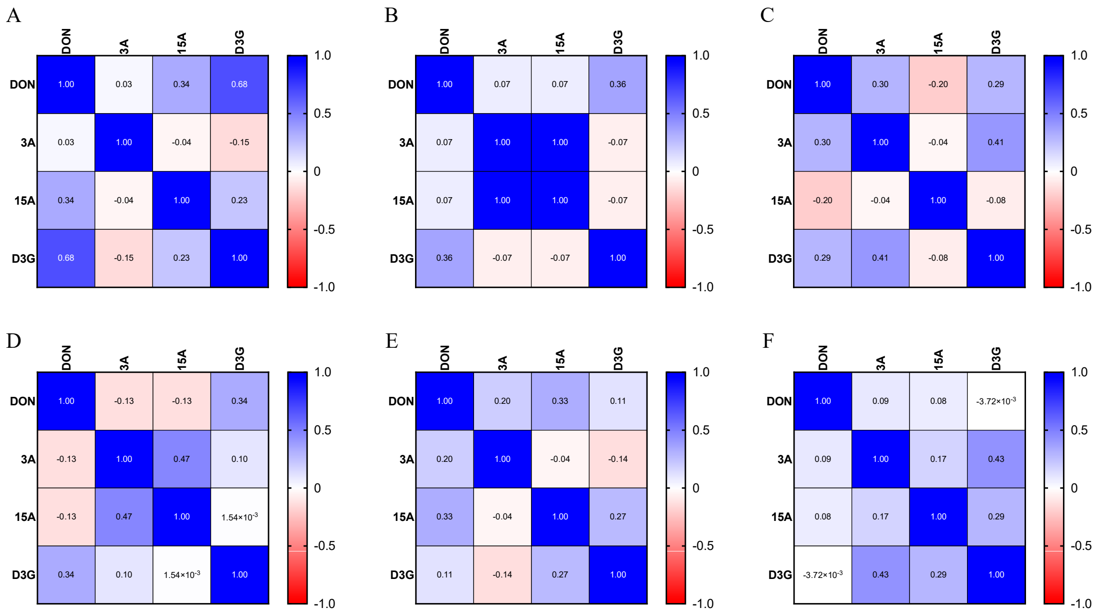

Figure S1. Correlation coefficient of detected mycotoxins in different cereals and their products. A: wheat flour; B: noodles; C: fermented products; D: beer; E: processed products; F: coarse grains.
